# Up-regulated expression of E2F2 is necessary for p16INK4a-induced cartilage injury

**DOI:** 10.1186/s12891-018-2253-x

**Published:** 2018-09-15

**Authors:** Xinnan Bao, Xinyu Hu

**Affiliations:** 1 Department of Orthopedics, The First People’s Hospital of Changzhou, No.185 Juqian Street, Changzhou, Jiangsu Province 213003 China; 2 Orthopedic Trauma Department, The First People’s Hospital of Changzhou, No.185 Juqian Street, Changzhou, Jiangsu Province, 213003 China

**Keywords:** p16INK4a, E2F2, Senescence-associated secretory phenotype (SASP), Osteoarthritis (OA)

## Abstract

**Background:**

Cartilage degradation would result in osteoarthritis (OA). p16INK4awas found in some age-related diseases. In this study, we aimed to determine the role of p16INK4a during OA and to investigate the underlying mechanisms.

**Methods:**

Enzyme-linked immunosorbent assay (ELISA) was performed to test the activity of senescence-associated secretory phenotype (SASP). Real-time PCR (RT-PCR) and Western blot were used to determine the expressions of target genes.

**Results:**

The increased expressions of p16INK4a and E2F2 were accompanied with cartilage degradation induced by IL-1β. Over-expression of p16INK4a enhanced the secretion of SASP markers (TGFβ, IL-6, IL-8, IL-1α, MMP3 and MMP13), reduced the expression of type II procollagen (COL2A1).Thus, the over-expression of p16INK4a lead to cartilage injury. Moreover, we found that the expression of E2F2 was enhanced in p16INK4a over-expression group, and that cartilage injury caused by p16INK4a was alleviated by depleting E2F2.

**Conclusions:**

p16INK4a was up-regulated during the cartilage injury in OA. p16INK4a promoted cartilage injury by increasing the expression of E2F2. Thus, this study extends the molecular regulation network for understanding pathological progression of OA, and provides potential therapeutic target for OA.

## Background

Osteoarthritis (OA) is one of the most common chronic diseases among aged population [1, 2]. Various factors, for example, abnormal joint development, joint injury, overweight, inherent factor and aging, contribute to the pathophysiology of OA [3, 4]. Although OA and aging are not inter-related, the onset and progression of the former is closely related to the later [5]. During the process of aging, senescence-associated secretory phenotype (SASP), which includes growth factors (such as TNF-β), pro-inflammatory cytokines (such as IL-6, IL-8, IL-1α) and matrix remodeling regulatory metalloproteases (such as MMP1 and MMP13) [6], expressed highly. SASP is able to induce inflammation that may lead to low-grade chronic inflammation and invovled in degenerative disorders including OA [7–10]. Viewed from the molecular perspective, OA is an outcome of cartilage degradation. The hallmark event in OA is the extracellular matrix degradation of articular cartilage [11]. Type II procollagen (COL2A1) helps maintain skeletal structure of cartilage [12]. The degradation of COL2A1 in cartilage matrix is critical in initiating cartilage degradation. So far, OA remains difficult to be treated, and the treatment strategies are largely restricted to symptom management [13]. Thus, preventing destruction of COL2A1 and the secretion of SASP may be helpful to delay the progression of OA.

The transcript of p16INK4a derives from alternative splicing of *INK4a/ARF* [14]. By binding to CDK4 and CDK6 and repressing phosphorylation of pRb, p16INK4a is well known as a cell cycle regulator [15]. Moreover, the increased expression of p16INK4a is often accompanied with cell senescence [16, 17].In addition, dysregulation of p16INK4a is common among human cancers [14, 18]. Researchers suggested that p16INK4a may participate in tumor cell escape from senescence [19, 20]. E2F2 is also a transcription factor that belongs to E2F family. Similar to p16INK4a, E2F2 takes part in cell cycle regulation [21, 22]. It has been reported that E2Fs could be released from pRb and could promote the G1/S. In addition, E2F2 can also maintain quiescence by repressing cell cycle regulators [21]. Researchers have proved that the transfection of E2F decoy oligodeoxynucleotides was helpful in preventing the generation of MMP-1, IL-1β and IL-6 [23]. As an apparent increase of E2F2 has been observed by researchers in rheumatoid arthritis (RA) synovial tissues [24], thus, it can be speculated that p16INK4a and E2F2 may participate in the progression of OA.

The aim of this study was to investigate the potential roles of p16INK4a and E2F2 in OA and to examine possible relations between p16INK4a and E2F2 in OA. The current study would expand the current understanding on pathophysiology of OA and provide promising drug target candidates for treating OA.

## Methods

### Cell culture

Human chondrocytes (#4650, ScienCell, USA) were cryopreserved at P0 and delivered frozen. The cells were cultured in DMEM/F-12 (Gibco, USA) containing 10% FBS at 37 °C. The medium was supplemented with 100 U/ml and penicillin/streptomycin. As previously described [25–27], recombinant IL-1β (R&D Systems) (10 ng/ml) was used to induce cartilage injury for 48 h. Experiments were performed independently for at least 3 times.

### Cell transfection

The cells (1.0 × 10^5^ cells per well) were seeded into 24-well plate. Prior to transfection, the cells have been starved overnight. pCMV-HA vector was a gift from Christopher A Walsh (Addgene plasmid #32530), pCMV-p16 INK4A was a gift from Bob Weinberg, (Addgene plasmid # 10916) and pCMV-HA-E2F2 was a gift from KristianHelin (Addgene plasmid # 24226) [28, 29]. The E2F2 siRNA (MBS8214676) and siRNA negative control (MBS8241404) were purchased from MyBio Source. The plasmid was transfected into the cells using Lipo 3000 Reagent (Life Science, USA). After being transfected for 6 h, the cells were maintained in fresh medium supplemented with 10% FBS and then prepared for the subsequent experiments.

### Cell proliferation assay

Cells (4000 cells/well) were plated into 24-well tissue culture plates (Corning Inc., Corning, NY). Cell proliferation was determined by using sulphonatedtetrazolium salt, and 4-[3-(4-iodophenyl)-2-(4-nitrophenyl)-2H-5-tetrazolio]-1 and 3-benzene disulphonate (WST-1) cell counting kits (Beyotime, China), following the manufactory’s instructions. The OD at 450 nm was read using a microplate reader (Biorad, USA).

### Enzyme-linked immunosorbent (ELISA) assay

The cells were harvested and centrifuged at 3000 g at 4 °C for 10 min. Following the manufacturer’s protocol, the levels of SASP markers TGFβ, IL-6, IL-8, IL-1α, MMP3 and MMP13 in the collected supernatants were examined using ELISA kits (R&D Systems). The absorbance was read at 405 nm using a micro-plate reader (Bio-rad, USA).

### Real-time PCR

Total RNA was isolated from cells using Trizol regent (Life Science) following the manufacturer’sprotocol. cDNA was reversed from total RNA using PrimeScript™ II 1st Strand cDNA Synthesis Kit (Takara, Japan). Amplification of the target genes from cDNA was performed using SYBR Green real-time PCR Master Mix (ToYoBo, Japan) under the conditions as follows: at 95 °C for 10 s, 40 cycles at 95 °C for 5 s and at 60 °C for 30 s. The primers used for RT-PCR were as follows:

p16INK4a sense: 5′- GCGGG GAGCAGCATGGAGC-3′;

p16INK4a anti-sense: 5′- CCGAATAGTTACG GTCG-3′;

E2F2 sense:5′-CCTTGGAGGCTACTGACAGC-3′;

E2F2antisense: 5′-CCACAGGTAGTCGTCCTGGT-3′;

Col II sense: 5′-CAATCCAGCAAACGTTCCCA-3′;

Col II antisense: 5′-CAGGCGTAGGAAGGTCATCT-3′;

Cdc6 sense: 5′- CAGCTGTTGAACTTCCCACC-3′;

Cdc6 antisense: 5′- GCTCTCCTGCAAACATCCAG-3′;

MCM6 sense: 5′-CCGAAATCCAGTTTGTGCCA-3′;

MCM6 antisense: 5′-TGCTAAGCTTGGAGACGTCA-3′;

β-actin sense: 5’-CTAAGGCCAACCGTGAAAAG-3′;

β-actin antisense: 5’-AACACAGCCTGGATGGCTAC-3′.

### Western blot

The cells were collected using cell lysissolution (Sigma) and denatured at 100 °C for 5 min. The protein concentrations were tested using bicinchoninic acid (BCA) Protein Assay Kit (Pierce, USA). Dodecyl sulphate polyacrylamide gel (SDS-PAGE) electrophoresis was performed to separate the proteins. Next, the proteins were transblotted onto nitrocellulose membranes (Amersham, USA). Primary antibodies were added after the membranes have been blocked with 5% not-fat milk. Then, the membranes were maintained at 4 °C overnight. Primary antibodies were as follows: anti-Col2A1 (1:8000, ab34712,abcam), anti-E2F2 (1:1000, ab65222), anti-p16INK4a (1:5000,ab108349) and anti-β-actin (ab8226,1:5000). Secondary antibodies (abcam) were incubated at room temperature for 2 h. The blot bands were developed with Enhanced chemiluminescence (Amersham).The density of bands was quantified using Quantity one 4.6.2.

### Statistics

Data were shown as mean ± Standard Deviation (SD). Student’s t test or one-way analysis of variance (ANOVA) following Dunett’s post hoc tests was used to compare result differences. *P* < 0.05 was considered as statistically significant.

## Results

### p16INK4a and E2F2 were up-regulated in Interleukin (IL)-1β- induced cartilage injury

**Fig. 1:**
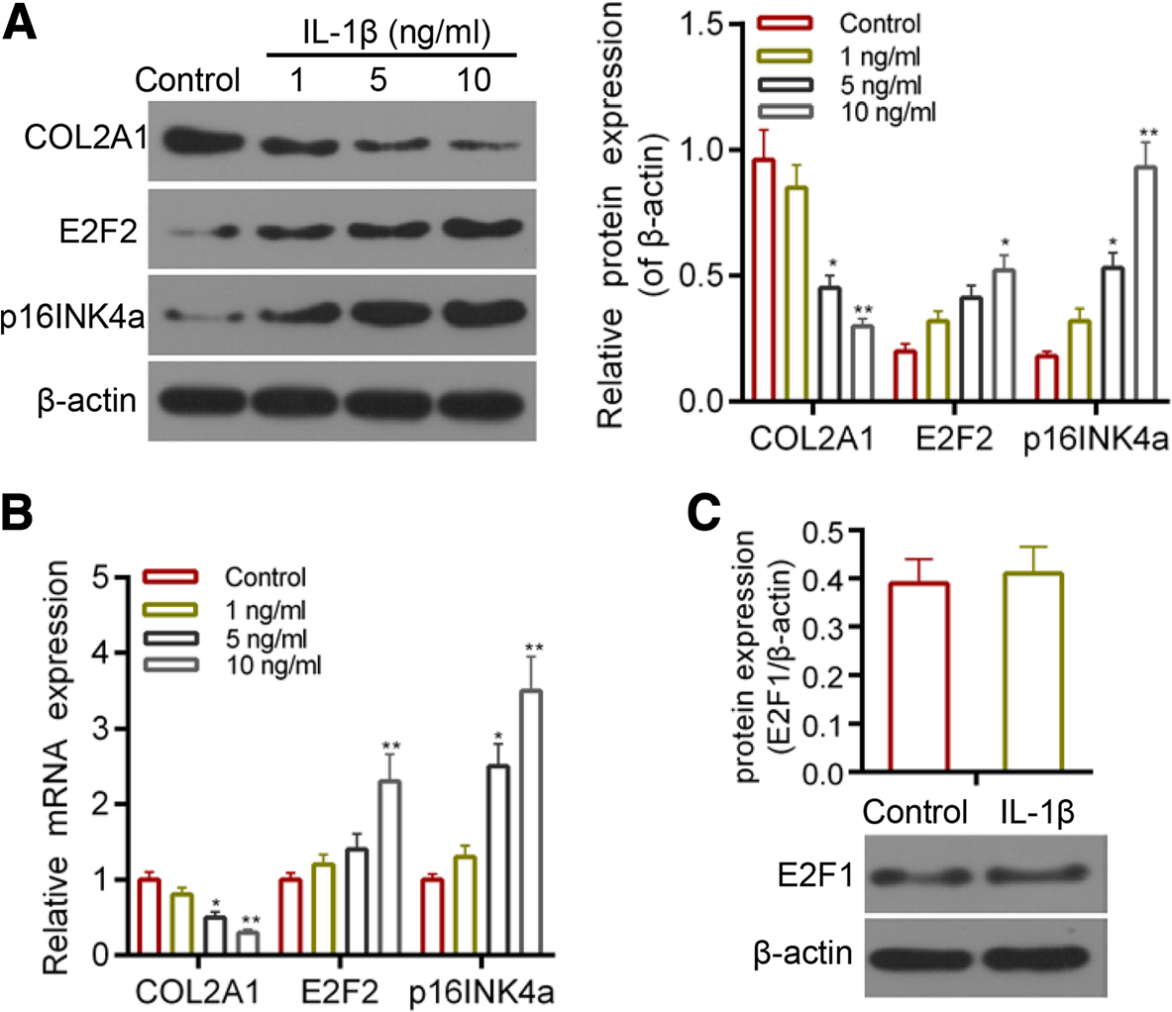
(**a**) Western blot assay for the expression of Col2A1, p16INK4a and E2F2. (**b**) Real-time PCR (RT-PCR) for the expression of Col2A1, p16INK4a and E2F2. (**c**) Western blot assay for the expression of E2F2 in the cells treated with 10 ng/ml IL-1β.*P < 0.05,**P < 0.01 vs. control. Data are shown as mean ± SD, *n* = 4

**Fig. 2:**
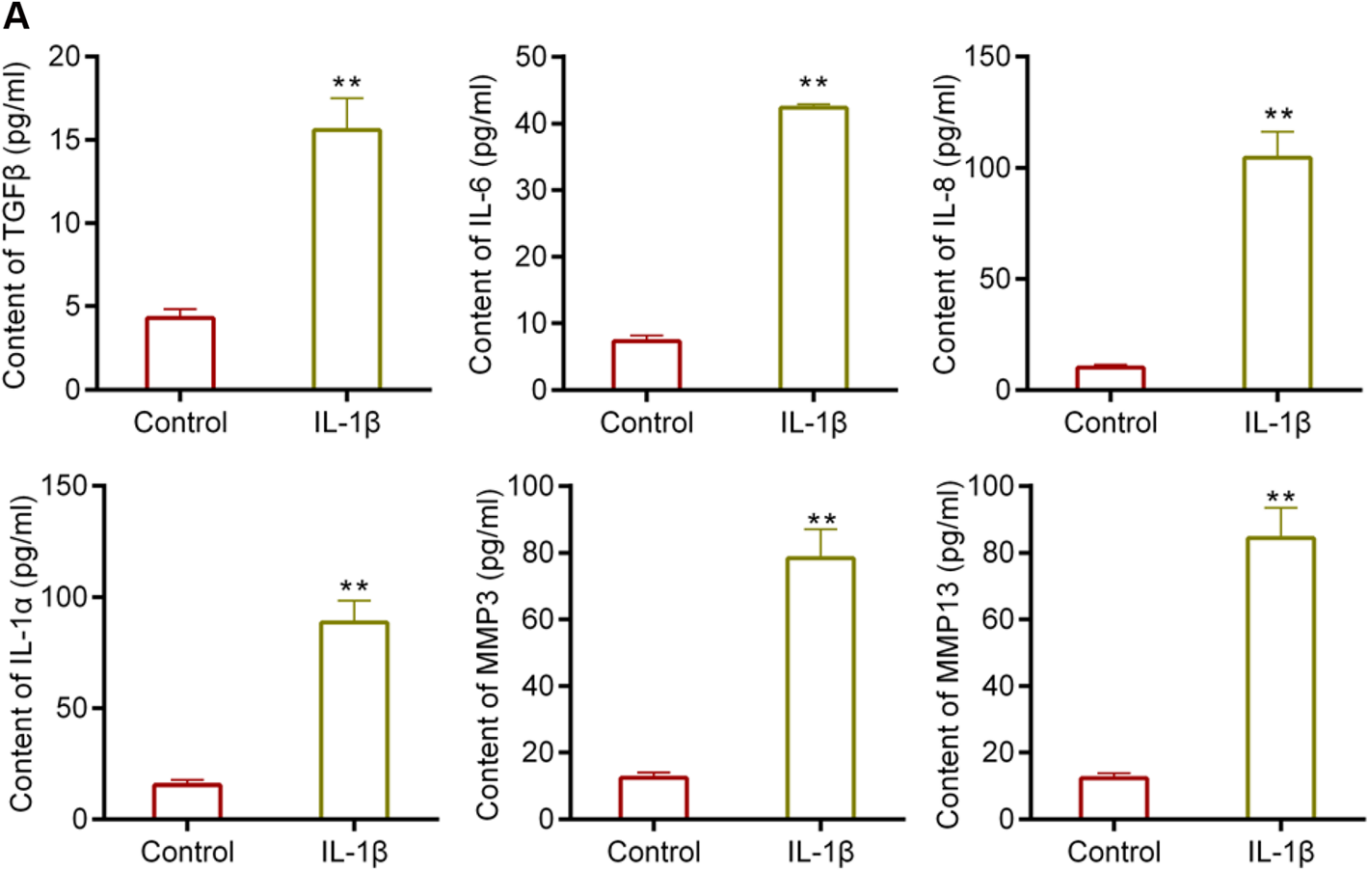
(**a**) Determination of the secretion of SASP markers, including TGFβ, IL-6, IL-8, IL-1α, MMP3 and MMP13, using ELISA assay. **P < 0.01 vs. control. IL-1β, cells were treated with IL-1β. Data were shown as mean ± SD, *n* = 5

*Interleukin* (IL)-1β, an important proinflammatory cytokine, contributes to the degradation of Col2A1 during OA [30, 31]. According to previous studies [32, 33], we detected the expressions of Col2A1, p16INK4a and E2F2 in the presence of 1, 5, 10 ng/ml IL-1β. We found that the expression of Col2A1 was decreased by IL-1β, while the expression of p16INK4a and E2F2 was increased by IL-1β. The effect of IL-1β was strongest at the 10 ng/ml (Fig. 1a-b). Thus, we selected 10 ng/ml IL-1β to treat the chondrocytes. In addition, the expression of E2F1 remains stable under the treatment of IL-1β (Fig. 1c). As shown in Fig. 2a, the secretion of SASP markers, which included TGFβ, IL-6, IL-8, IL-1α, MMP3 and MMP13 was induced by the treatment of 10 ng/ml IL-1β.

### The effect of p16INK4a over-expression on cartilage injury

**Fig. 3:**
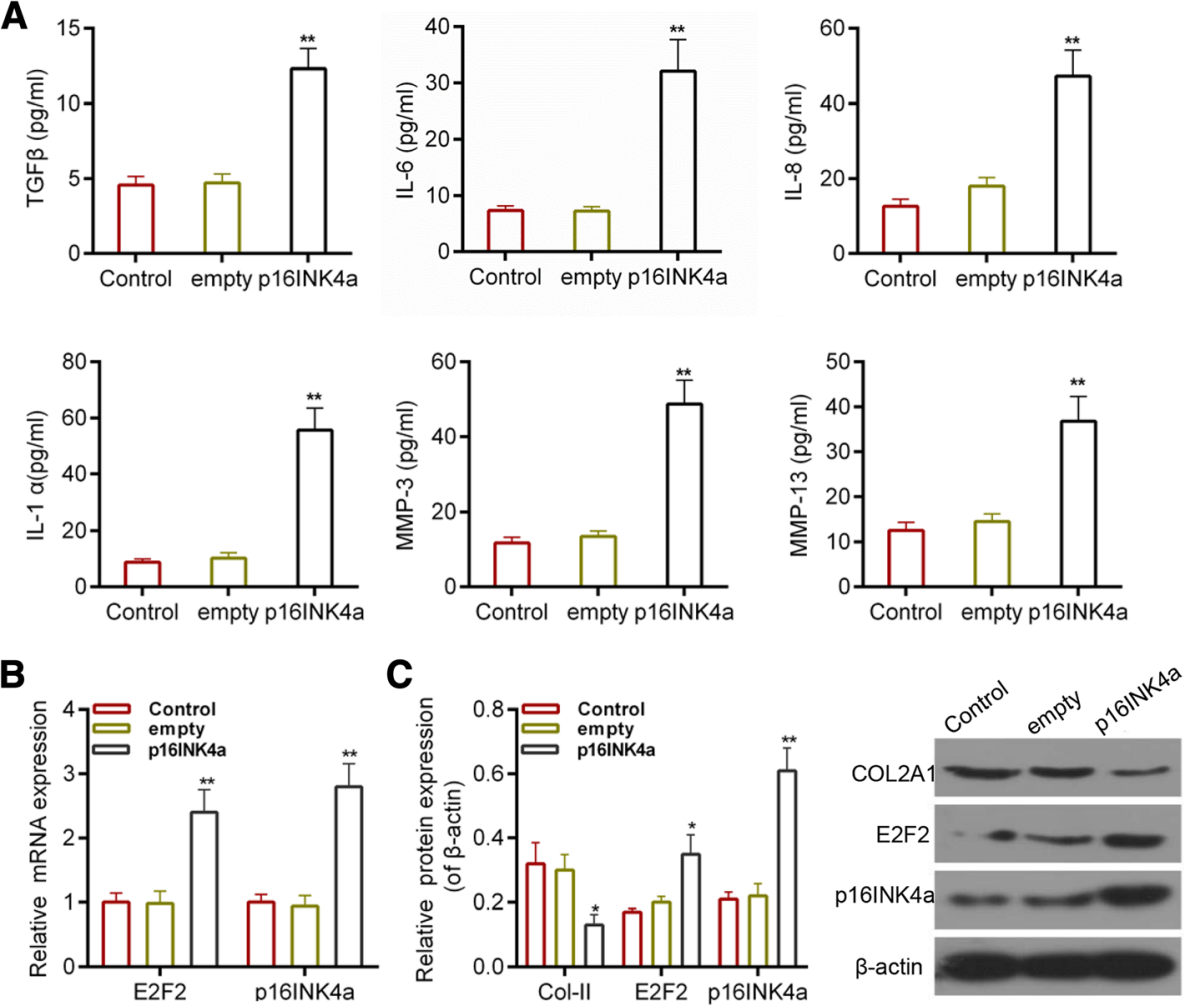
(**a**) ELISA assay for the secretion of SASP markers after the over-expression of p16INK4a. (**b**) RT-PCR for detecting the expressions of Col2A1, p16INK4a and E2F2. (**c**) The expressions of Col2A1, p16INK4a and E2F2 by Western blot assay. *P < 0.05 and **P < 0.01 vs. empty. Empty, cells were transfected with over-expression empty vector; p16INK4a, cells were transfected with p16INK4a over-expression vector. Data were shown as mean ± SD, n = 5

p16INK4a has been recognized as a senescent contributor in various tissues [34]. The expression of SASP markers was determined in order to investigate the role of p16INK4a in cartilage injury. The results showed that over-expression of p16INK4a enhanced the secretion of TGFβ, IL-6, IL-8, IL-1α, MMP3 and MMP13 (Fig. 3a), and that the expression of Col2A1 was also reduced by over-expression of p16INK4a. Interestingly, the expression of E2F2 was higher in p16INK4a group than that in control group (Fig. 3b-c).

### E2F2 was necessary for the cartilage injury caused by over-expression of p16INK4a

**Fig. 4:**
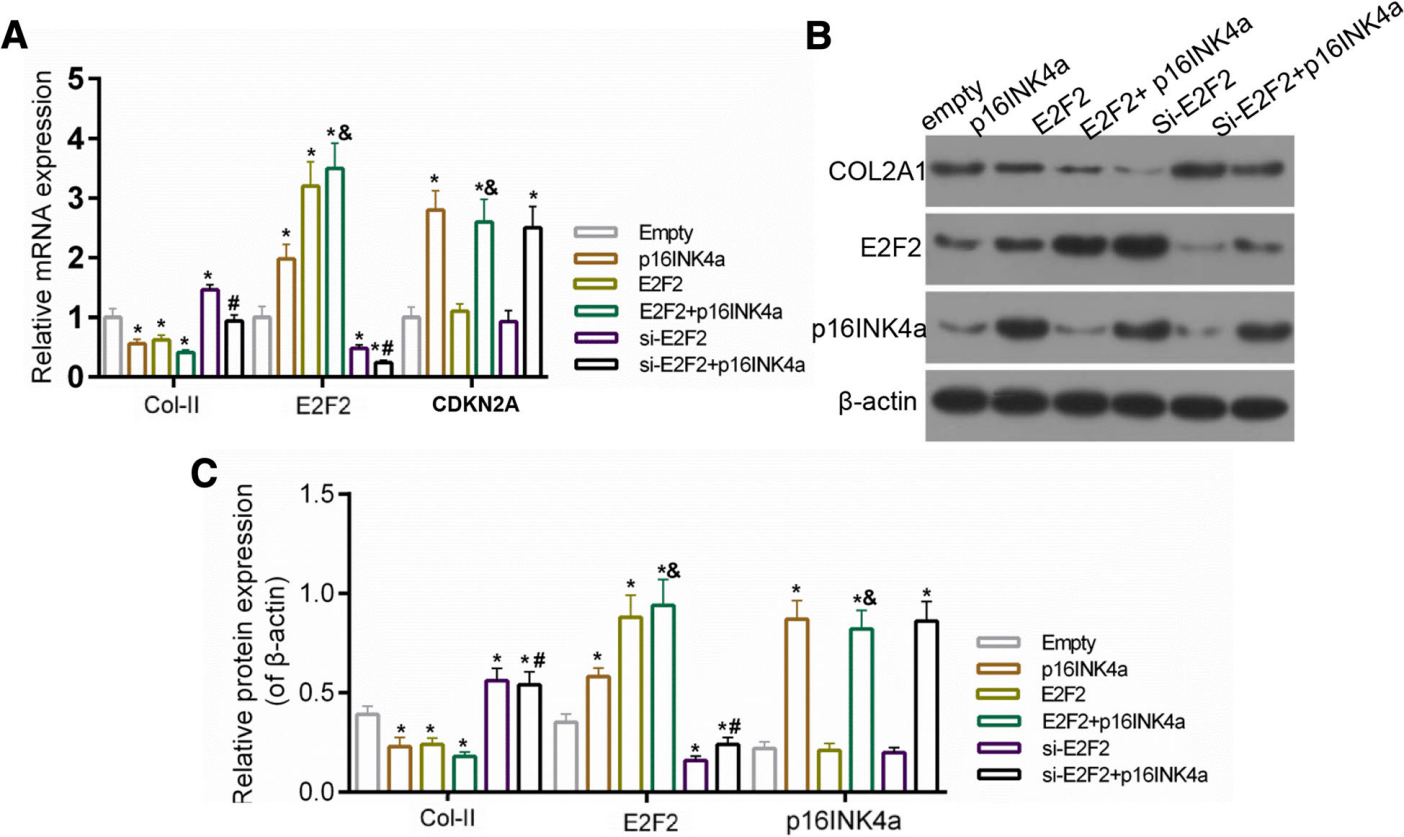
(**a**) The expression of Col2A1, p16INK4a and E2F2 determined by RT-PCR. (**b**-**c**) The expressions of Col2A1, p16INK4a and E2F2 determined by Western blot assay. Empty group, cells were transfected with over-expression empty vector and siRNA negative control; p16INK4a group, cells were transfected with p16INK4a over-expression vector and siRNA negative control; E2F2 group, cells were transfected with E2F2 over-expression vector and siRNA negative control; p16INK4a + E2F2 group, cells were over-expressed with E2F2 and p16INK4a and siRNA negative control; si-E2F2 group, cells were transfected with si-E2F2 and over-expression empty vector; p16INK4a + si-E2F2 group, cells were transfected with si-E2F2 and p16INK4a. *P < 0.05 vs. empty, #*P* < 0.05 vs. p16INK4a. Data were shown as mean ± SD, n = 4

**Fig. 5:**
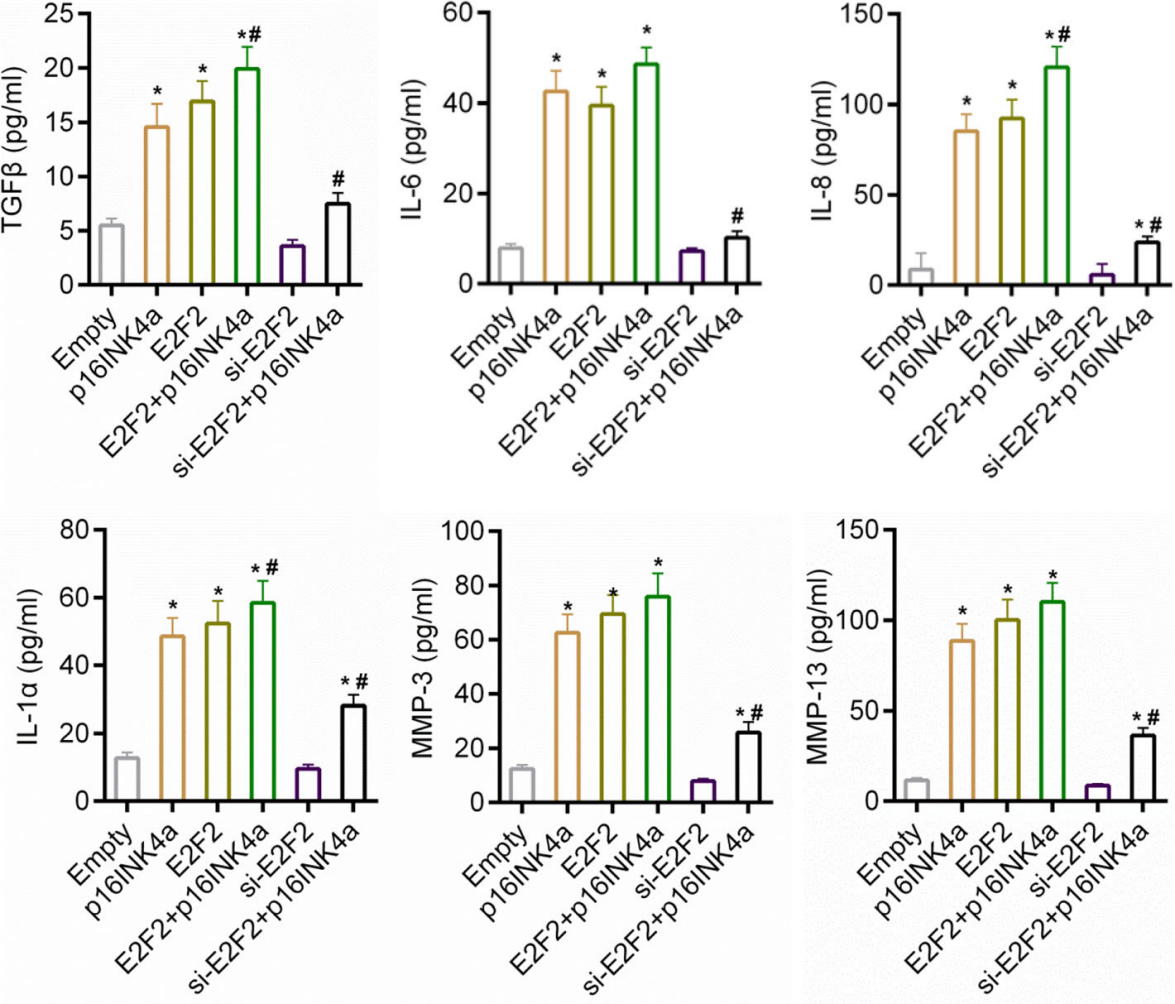
ELISA assay for SASP markers. Empty group, cells were transfected with over-expression empty vector and siRNA negative control. p16INK4a group, E2F2 indicated the over-expressions of p16INK4a and E2F2 respectively. Si-E2F2 indicated the depletion of E2F2. *P < 0.05 vs. empty. ^#^*P* < 0.05, vs. p16INK4a. Data were shown as mean ± SD, n = 5

The effect of E2F2 on cartilage injury was examined in order to further study the potential relation between p16INK4a and E2F2. As shown in Fig. 4, compared to control group, the over-expressions of both p16INK4a and E2F2 reduced the expression of Col2A1, which was then increased by the depletion of E2F2. Moreover, the expression of Col2A1 was found to be lower in p16INK4a + si-E2F2 group than that in p16INK4a group. Nevertheless, the expression of Col2A1 was slightly reduced in p16INK4a + E2F2 group, compared to that in p16INK4a group. Furthermore, results from ELISA showed that secretion of SASP markers (TGFβ, IL-6, IL-8, IL-1α, MMP3 and MMP13) was increased in p16INK4a and E2F2 groups, while such a secretion was repressed in si-E2F2 group. Moreover, compared to that in p16INK4a group, the secretion of SASP markers was inhibited in p16INK4a + si-E2F2 group (Fig. 5).

### The effect of p16INK4a over-expression on cell proliferation of chondrocytes

**Fig. 6:**
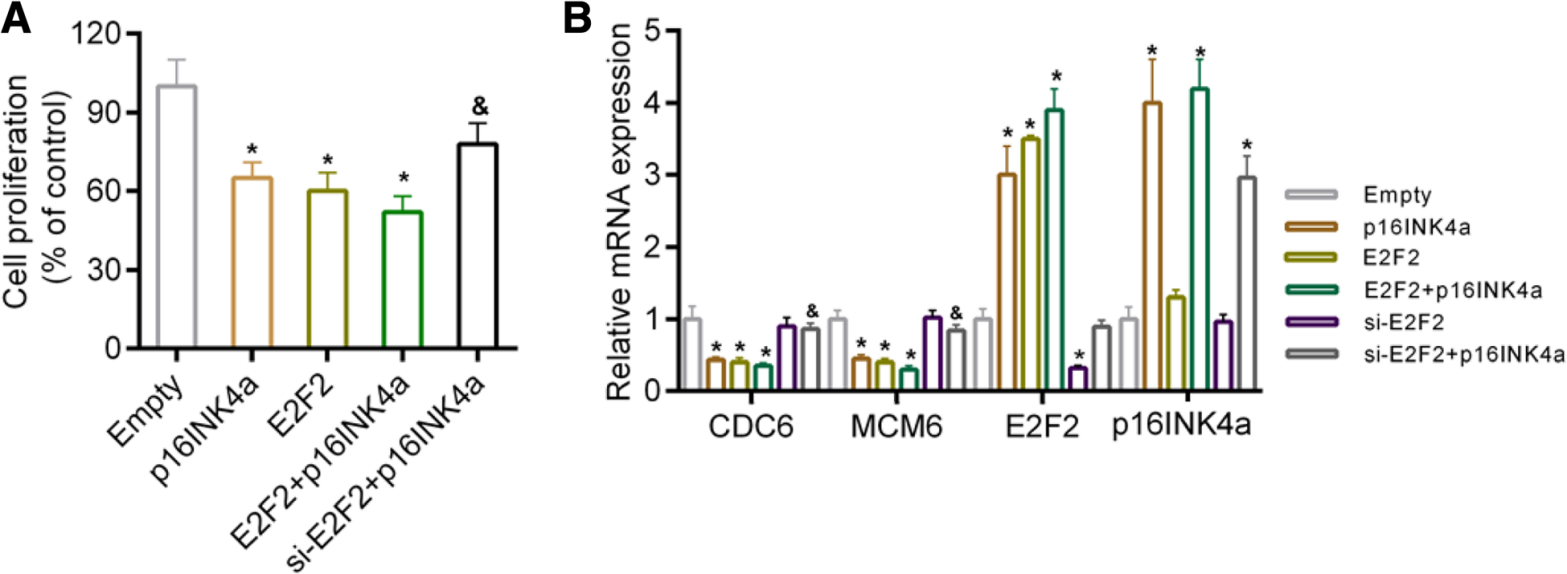
(**a**) Cell proliferation was detected by WST-1 cell counting kit. *P < 0.05 vs. control. Data were shown as mean ± SD, *n* = 5. (**b**) The expressions of CDC6, MCM6, p16INK4a and E2F2 determined by RT-PCR. Data were shown as mean ± SD, *n* = 4.*P < 0.05 vs. empty.^&^*P* < 0.05, vs. p16INK4a

Cell proliferation was determined by using the WST-1 cell counting kit. We found that transfection of p16INK4a or E2F2 resulted in a significant inhibition of cell proliferation. The down-regulation of E2F2 was observed to recover the proliferation of cells that have been transfected with p16INK4a (Fig. 6a). In addition, the mRNA expression of cell cycle-specific genes was detected, and the data showed that transfection of p16INK4a or E2F2 inhibited the expressions of CDC6 and MCM6, and that the down-regulation of E2F2 rescued the expressions of CDC6 and MCM6 in the cells that have been transfected with p16INK4a (Fig. 6b).

## Discussion

Tissue deconstruction is a consequence of aging. The integrity and function loss in senescent cells are caused by chronic inflammation and remodel of extracellular matrix [35, 36]. OA is a degenerative disease accompanied with progressive cartilage degradation. Aging is one of the most significant risk factors in OA [1, 2]. Although the mechanisms underlies OA awaits to be fully understood, the cartilage integrity is believed to be affected by inflammatory cytokines and matrix remodeling.

In this study, based on a previous study [37], IL-1β was used to induce the cartilage degradation so as to establish a model of OA. The secretion of SASP markers TGFβ, IL-6, IL-8, IL-1α, MMP3 and MMP13 was found to be increased in response to IL-1β treatment. Moreover, the expression of Col2A1, an index of cartilage degradation [38], was reduced in IL-1β group in comparison to that in control group. p16INK4a is realted to age-related diseases [39]. As expected, the expression of p16INK4a was strongly induced by IL-1β. Consistent to our results, a previous study has also pointed out that SASP was exhibited in p16INK4a-positive cells [36]. A study has also reported that expression of p16INK4a was a biomarker of chondrocyte aging and was correlated with several SASP transcripts even though the loss of p16 did not affect the expression of SASP in mouse [40].These results revealed that the expression of p16INK4a was positively related to the secretion of SASP during cartilage injury. However, SASP can also be restrained by p16INK4a under some conditions [41]. The different effect of p16INK4a on SASP may be caused by different cell contexts. In addition, as a transcription factor, E2F2 was increased in RA synovial tissues [24]. Thus, we determined the expression of E2F2 in IL-1β-treated cells, and found a higher expression of E2F2 in IL-1β group in comparison to that in control group. As a family member of E2F2, E2F1 may have an overlapping function with E2F2. Nevertheless, the expression of E2F1 was not affected by IL-1β.

It has been proved that p16INK4a was able to inhibit cell cycle by targeting CDK4/6, and therefore maintaining the activity of retinoblastoma (pRb) that could control cell fate decision of chondrocytes [42–44]. E2F family members can be released from pocket protein members (retinoblastoma, p107 and p130) and can promote the G1/S progression [45, 46]. The effect of p16INK4a over-expression was examined in this study. Our results showed that secretion of SASP was promoted by over-expressing p16INK4a. Furthermore, the expressions of Col2A1 and E2F2 were higher in p16INK4a group in comparison to those in control. Taken together, the cartilage injury was expanded by over-expressing p16INK4a. In addition, the increased expression level of E2F2 may exacerbate cartilage injury. Consistently, some recent studies pointed that E2F2 functioned as a repressor of transcription and an inhibitor of cell proliferation in some cell context [47, 48]. Therefore, it is possible that E2F2 may interact with other proteins or factors functioning as a negative transcriptional regulator [49].

Subsequent investigations were performed to further determine whether E2F2 was necessary for cartilage injury caused by p16INK4a*.* Our results indicated that cartilage injury was induced by over-expressing p16INK4a and E2F2, by increasing the secretion of SASP markers and by repressing the expression of Col2A1. Moreover, we detected the expressions of cell cycle specific genes (CDC6 and MCM6). CDC6 is essential for the assembly of MCM complex in DNA replication and that MCM complex assembly is necessary for cells to enter S-phase [50].The cell proliferation and the expressions of CDC6 and MCM6 were inhibited by over-expressing p16INK4a or E2F2. The down-regulation of E2F2 in the cells transfected with p16INK4a was observed to recover the cell proliferation and the expressions of CDC6 and MCM6. However, according to previous studies, the peak expressions of CDC6 and MCM6 are in G1/S during DNA replication and are dependent on E2F [51–53]. Thus, it is possible that over-expressing E2F2 might prevent cell cycle progression through competitive inhibition of E2F2 transcriptional activity (though the mechanism underlying such a competitive inhibition was not clear). Taken together, the depletion of E2F2 alleviated cartilage injury caused by p16INK4a, suggesting that E2F2 was necessary for p16INK4a-induced OA progression. Nevertheless, the co-expression of p16INK4a and E2F2 increased the secretion of TGFβ and IL-8 in comparison to over-expressing p16INK4a alone. However, no significant synergistic action of p16INK4a and E2F2 was observed. A previous study has shown that other signals or pathways, for example, p38 MAPK and extracellular signal-regulated kinase (ERK) signaling, also took part in cell senescence [54]. This may be explained by the fact that cartilage degradation is controlled by many other signals and regulators [55, 56]. Therefore, the molecular mechanism of cartilage degradation still remains to be further investigated.

In addition, microRNAs have also been used in diagnosis of OA. For instance, Researchers have proved that p16INK4a could be regulated by miR-24 during matrix remodeling in OA [57]. Therefore, it would be helpful for the diagnosis and molecular treatment of OA to explore the same type of regulators of p16INK4a during OA. Although it can be concluded that E2F2 is a downstream target of p16INK4a, unfortunately, within the scope of this study, we were unable to provide a specific explanation about how p16INK4a regulated E2F2. Thus, it would be interesting to study whether SASP markers and/or Col2A1can be directly regulated by E2F2. It is beneficial to further illustrate the molecular pathological of cartilage degradation during OA.

## Conclusions

In summary, this study demonstrated that over-expression of p16INK4a promoted cartilage injury. Over-expression of p16INK4a increased the secretion of SASP markers and reduced the expression of Col2A1. Moreover, the expression of E2F2 was necessary for p16INK4a-induced cartilage injury. Overall, this study determined the role of p16INK4a/E2F2 in OA, and such a result helped provide therapeutic targets for treating OA.
